# Sequential or Concomitant Inhibition of Cyclin-Dependent Kinase 4/6 Before mTOR Pathway in Hormone-Positive HER2 Negative Breast Cancer: Biological Insights and Clinical Implications

**DOI:** 10.3389/fgene.2020.00349

**Published:** 2020-04-15

**Authors:** Giulia Occhipinti, Emanuela Romagnoli, Matteo Santoni, Alessia Cimadamore, Giulia Sorgentoni, Monia Cecati, Matteo Giulietti, Nicola Battelli, Alessandro Maccioni, Nadia Storti, Liang Cheng, Giovanni Principato, Rodolfo Montironi, Francesco Piva

**Affiliations:** ^1^Department of Specialistic Clinical and Odontostomatological Sciences, Polytechnic University of Marche, Ancona, Italy; ^2^Oncology Unit, Macerata Hospital, Macerata, Italy; ^3^Section of Pathological Anatomy, School of Medicine, United Hospitals, Polytechnic University of the Marche Region, Ancona, Italy; ^4^Direzione Area Vasta 3, Macerata, Italy; ^5^Direzione Sanitaria Azienda Sanitaria Unica Regionale, Ancona, Italy; ^6^Department of Pathology and Laboratory Medicine, Indiana University School of Medicine, Indianapolis, IN, United States

**Keywords:** breast cancer, estrogen receptor, cyclin-dependent kinases (CDKs), CDK inhibitors, mechanisms of resistance

## Abstract

About 75% of all breast cancers are hormone receptor-positive (HR+). However, the efficacy of endocrine therapy is limited due to the high rate of either pre-existing or acquired resistance. In this work we reconstructed the pathways around estrogen receptor (ER), mTOR, and cyclin D in order to compare the effects of CDK4/6 and PI3K/AKT/mTOR inhibitors. A positive feedback loop links mTOR and ER that support each other. We subsequently considered whether a combined or sequential inhibition of CDK4/6 and PI3K/AKT/mTOR could ensure better results. Studies indicate that inhibition of CDK4/6 activates mTOR as an escape mechanism to ensure cell proliferation. In literature, the little evidence dealing with this topic suggests that pre-treatment with mTOR pathway inhibitors could prevent or delay the onset of CDK4/6 inhibitor resistance. Additional studies are needed in order to find biomarkers that can identify patients who will develop this resistance and in whom the sensitivity to CDK4/6 inhibitors can be restored.

## Introduction

Breast cancer presents a significant health burden worldwide according to the International Agency for Research on Cancer^1^. About 75% of all breast cancers are hormone receptor-positive (HR+). HR+ cancers express estrogen receptors (ER) and/or progesterone receptors (PgR). Such tumors are basically dependent on the ER-related signaling pathway in order to grow and survive. The ER-related signaling pathway regulates various cellular functions, such as apoptosis and cell proliferation as well as angiogenesis. HR+ cancers take advantages of the ER pathway in order to promote cancer development, growth, and progression (Platet et al., 2004). This has led to the development of therapeutic agents targeting the estrogen signaling pathway, i.e., aromatase inhibitors (AIs; such as exemestane, anastrozole and letrozole), selective down-regulators of ERs (fulvestrant), selective modulators of ERs (tamoxifen). This endocrine therapy has become the treatment of choice for breast cancers that are HR+ (Milla-Santos et al., 2003; Di Leo et al., 2014).

The efficacy of such a therapeutic approach is however limited. This, due to the high rate of pre-existing as well as acquired resistance (Osborne and Schiff, 2011; De Marchi et al., 2016), has led to a proportion of patients failing to respond to endocrine therapy. Resistance to endocrine therapy is an important problem from the clinical point of view.

### Target Therapy: mTOR Inhibitors

Information on the PI3K/AKT/mTOR pathway has led to the development of mTOR inhibitors (Hosford and Miller, 2014), including Everolimus. In 2012 this drug was approved for the treatment of ER+/HER2- metastatic cancer following progression on previous non-steroidal AI.

Effectiveness of combining endocrine therapy with mTOR inhibitors has been tested in several trials, including the TAMRAD trial, a randomized phase 2 trial of tamoxifen with or without everolimus in postmenopausal women with AI-resistant, ER+, advanced cancer. The combination arm was associated with a significantly better progression-free survival (PFS; 4.5 vs. 8.6 months) and overall survival (OS) (Bachelot et al., 2012). BOLERO-2 is a phase 3 trial of exemestane combined with either everolimus or placebo. This was conducted in postmenopausal women with advanced ER+/HER2- cancer resistant to either letrozole or anastrozole. The study showed an improvement in PFS (3.2 months in the placebo/exemestane arm vs. 7.8 months in the everolimus/exemestane arm) (Yardley et al., 2013). Moreover, everolimus resulted to be effective in combination with fulvestrant in AI-resistant luminal metastatic cancer (NCT01797120) or with letrozole as first-line treatment in postmenopausal patients with luminal metastatic cancer (NCT01698918).

### Target Therapy: CDK 4/6 Inhibitors

The advent of cyclin-dependent kinases (CDKs) 4/6 inhibitor-based combination therapies represents a challenge for breast cancer treatment. The development of inhibitors of CDK4 and CDK6 has changed the perception of CDKs as therapeutic targets in breast cancer following “underwhelming results and unacceptable toxicity were seen with pan-CDK inhibitors such as flavopiridol (alvocidib) in the early 2000s” (Fry et al., 2004; Toogood et al., 2005; Hosford and Miller, 2014; Asghar et al., 2015). Palbociclib is an orally active pyridopyrimidine, highly selective reversible inhibitor of CDK4 and CDK6. The effect of palbociclib is linked on the presence of a functional retinoblastoma protein (Rb), whereas there is no effect on Rb-deficient cells (Kim et al., 2014). On the other hand, abemaciclib, and ribociclib are selective small molecule reversible inhibitors of CDK4/6. It is hypothesized that the selectivity of palbociclib, abemaciclib, and ribociclib is due to the specific interactions with aminoacids of the ATP-binding site in the catalytic cleft of CDK4 and CDK6 (Asghar et al., 2015).

At present, no studies have directly compared the safety and efficacy of the two sequences CDK4/6 inhibitor/everolimus vs. everolimus/CDK4/6 inhibitor. Our computational study aims to show the inhibition of the pathways by the CDK 4/6 inhibitors and everolimus and the biological rational of sequencing or combining these two therapeutic strategies. Literature search has been performed in PubMed by using combinations of specific search terms, such as “breast,” “resistance,” “CDK4,” “CDK6,” “mTOR,” “PI3K,” “inhibitor,” or the corresponding drug names and their synonyms. We have selected research articles from 2001 focusing on ER+ or triple negative cancer cell lines, human ER+ breast tumor samples and patient derived xenograft models. Finally, some review articles, analyzing alterations in the PI3K/AKT/mTOR pathway in ER+ breast tumors associated with endocrine therapy resistance, where selected.

## Cell Cycle Signaling Pathway

Cell cycle is regulated by the CDK4/6-Rb-E2F axis. Several other factors strictly control this axis including cyclin D, INK4 family proteins (p15^INK4b^, p16^INK4a^, p18^INK4c^, and p19^INK4d^), p21^CIP1^, and p27^Kip1^ (Olmez et al., 2017).

**FIGURE 1:**
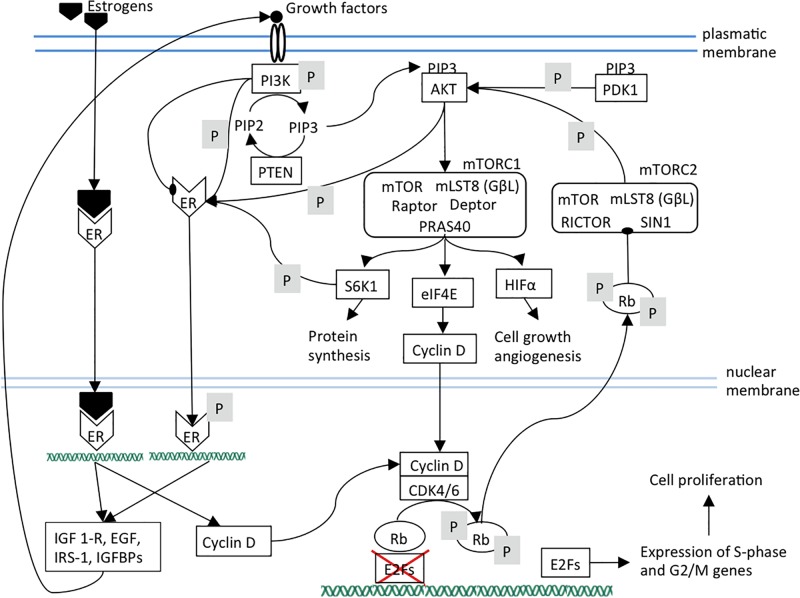
The ER, mTOR, and CDK4/6 pathways and their links are shown. Arrows indicate activation; arrows ending with oval tips indicate inhibition; P means phosphorylation. It can be observed the presence of positive feedback loops that maintain the three pathways. In particular, mTOR pathway components sustain estrogen pathway by activating ER independently of its ligands. Estrogen pathway sustains mTOR pathway by increasing the expression of its upstream receptors and ligands. Additionally, estrogen and mTOR pathways reinforce CDK4/6 pathway through cyclin D expression. It should be noted that PI3K can also inhibit ER. ER, estrogen receptor; PI3K, Phosphatidylinositol 3-kinase regulatory subunit alpha; PIP3, phosphatidylinositol (3,4,5)-trisphosphate; PIP2, phosphatidylinositol (4,5)-bisphosphate; PTEN, phosphatase and tensin homolog; AKT, serine/threonine kinase 1; PDK1, pyruvate dehydrogenase kinase 1; mTOR, mammalian target of rapamycin; mTORC1, mTOR complex 1; mTORC2, mTOR complex 2; mLST8, mammalian lethal with SEC13 protein 8, also known as G protein beta subunit-like (GβL); Raptor, regulatory-associated protein of mTOR; PRAS40, Proline-rich AKT1 substrate 1; Deptor, DEP domain-containing mTOR-interacting protein; RICTOR, Rapamycin-insensitive companion of mammalian target of rapamycin; SIN1, mammalian stress-activated protein kinase interacting protein 1; S6K1, ribosomal protein S6 kinase B1; eIF4A, eukaryotic initiation factor-4A; HIFα, hypoxia inducible factor 1 subunit alpha; Rb, retinoblastoma protein; CDK4, cyclin dependent kinase 4; CDK6, cyclin dependent kinase 6; E2F, transcription factor.

The activated cyclin D-CDK4/6 complex provokes full phosphorylation and functional inactivation of Rb. Rb active form consists in its hypo-phosphorylated status. In this way, active Rb acts as tumor suppressor primarily through binding and consequent suppression of the nuclear E2F family of transcription factors as well as by governing p27Kip1 stability partly through interacting with APC^*Cdh1*^; this causes cell cycle arrest in the transition from G1 to S phase. Hyper-phosphorylated Rb loses its suppressive role toward E2F in the nucleus leading to the expression of E2F target genes required for S-phase entry. In the cytoplasm, hyper-phosphorylated Rb binds Sin1, a component of mTORC2 complex, thus preventing AKT phosphorylation and mTORC1 activation (Zhang et al., 2016; Bonelli et al., 2017; Figure 1). mTOR signaling is activated by a variety of different signals such as EGF, IGF-1, amino acids or high cellular energy. Growth factors activate the phosphatidylinositol 3-kinase (PI3K) that phosphorylates PIP2 to PIP3. The increased amount of PIP3 allows the co-localization of the serine/threonine kinase AKT, phosphoinositide-dependent kinase-1 (PDK1) and mTORC2 to the plasma membrane. PDK1 and mTORC2 phosphorylate AKT leading to the complete AKT activation that, in turn, activates mTORC1 (Kim et al., 2017). mTORC1 complex (i) activates the transcription factor HIF1α that induces cell growth and angiogenesis, (ii) releases the translation factor eIF4E that enhances mRNA expression and export of genes as cyclin D, therefore favoring cell proliferation (Rosenwald et al., 1995; Michaloglou et al., 2018), (iii) activates the 40S ribosomal S6 kinase 1 (S6K1) that increases protein synthesis (Michaloglou et al., 2018).

Briefly, Rb is involved in two independent pathways. Hypo-phosphorylated Rb suppresses E2F1, inducing the cell cycle arrest at G1 phase. The hyper-phosphorylated Rb stops the AKT oncogenic signaling pathways through mTORC2 inhibition (Zhang et al., 2016).

Estrogens stimulate cell proliferation by increasing cyclin D expression, but also in an indirect way through mTOR. In fact, PI3K/AKT/mTOR cascade is also involved in ER pathway triggering since S6K1, AKT, and PI3K induce ER phosphorylation and activation independently to the estrogen presence (Campbell et al., 2001; Yamnik et al., 2009). Consequently, despite the estrogen deprivation, ER signaling can be activated resulting in endocrine therapy resistance. The trigger of the ER signaling pathway, both estrogen-dependent and independent, induces the transcription of genes (such as IGF1-R, EGF, IRS-1, IGFBPs) encoding for PI3K/AKT/mTOR pathway effectors resulting in a positive feedback loop (Miller et al., 2011; Ciruelos Gil, 2014; Figure 1). However, the interaction between these two pathways seems more complicated, since there could be even an inverse correlation of PI3K activation with ER expression levels. In a model of ER+ cancer, PI3K inhibition led to an increase of ER expression, whereas PI3K activation induced a decrease of ER expression level (Creighton et al., 2010). This evidence is supported by another study which demonstrated that in ER+ breast cancer models the PI3K inhibition enhanced the expression of ER at both mRNA and at protein levels. This, in turn, promoted the expression of the genes regulated by ER (Bosch et al., 2015).

The combination of mTOR or PI3K inhibitors to CDK4/6 inhibitor therapy have shown encouraging preliminary results in clinical trials, although PI3K inhibitors resulted to be quite toxic and poorly effective and, therefore, they are not yet approved for breast cancer (Hamilton and Infante, 2016; Cortes et al., 2017; Ballinger et al., 2018).

### Sequential Inhibition of CDK4/6 and mTOR

**FIGURE 2:**
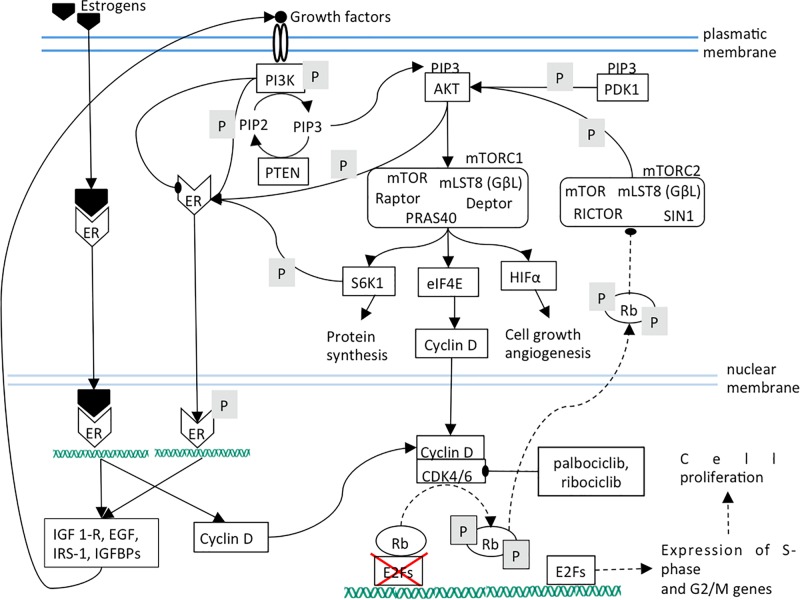
Dashed lines indicate a decreased interaction between two components or a diminished function. The effects of CDK4/6 inhibition are shown. Mainly, Rb phosphorylation and its translocation into cytoplasm are prevented.

**FIGURE 3:**
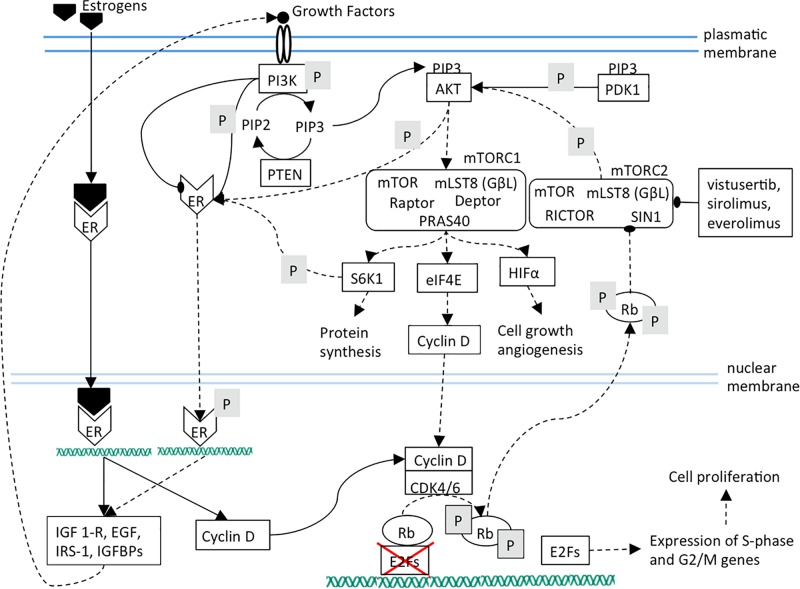
The effects of mTOR inhibition are shown. It can be noted that mTOR inhibition causes cell proliferation arrest by cyclin D diminished synthesis and ER target genes down-regulation.

The blockade of CDK4/6 activity by therapeutic inhibitors leads to Rb de-phosphorylation and to the subsequent strengthening of PI3K/AKT/mTOR pathway that plays a pivotal role in the control of cell growth, migration and metabolism (Cretella et al., 2018; Figure 2). mTORC2 inhibition lowers cell proliferation by weakening of mTORC1, cyclin D, Rb axis. Additionally, ligand-independent ER phosphorylation is reduced, leading to a lower expression of growth factors and receptors and therefore to a lower mTOR activation (Figure 3).

Recently, it has been showed that treatment with palbociclib alone for 24 h in TNBC (triple negative breast cancer) cell lines increases AKT activation. After palbociclib pre-incubation, the sequential combined treatment with PI3K inhibitors plus palbociclib was associated with G0/G1 cell cycle arrest in a greater percentage of cells. This sequential combined treatment determined a higher mTOR inhibition and Rb de-phosphorylation, in comparison with the single or simultaneous drug treatment. Notably, the sequential treatment induced a stronger impairment of the glucose metabolism. In fact, palbociclib treatment maintains Rb in its active form with the consequent repression of E2F transcription factor and down-regulation of the transcription factor c-myc, a direct target of E2F. In turn, c-myc down-regulation results in decreased expression of the GLUT-1 glucose transporter. The combination of palbociclib with BYL719 (PI3K inhibitor) enhanced these effects under normoxic and hypoxic conditions, since the inhibition of PI3K/mTOR signaling is also involved in glucose uptake decrease (Cretella et al., 2018).

The synergistic effect on cell cycle arrest and senescence of sequential treatment with palbociclib and PI3K/mTOR inhibitors was observed also in malignant pleural mesothelioma cell models (Bonelli et al., 2017).

### Combined Inhibition of CDK4/6 and mTOR

ER+ breast cancer frequently shows a hyperactivation of the mTOR pathway. Some studies have shown advantage from combining mTOR inhibitors with estrogen receptor blockade (Bachelot et al., 2012; Yardley et al., 2013). However, the precise relationship among ER signaling and mTOR pathways has not been clarified. Moreover, activation of CDK–Rb–E2F signaling has often been associated with endocrine resistance; for this reason, CDK4/6 inhibitors, such as palbociclib, are investigated in clinical trials in ER+ cancer. In two breast cancer cell lines, MCF-7 and ER+HCC-1428, the mTORC1/2 inhibitor vistusertib (AZD2014) caused decreased levels of cyclin D1 resulting in the hypo-phosphorylation of Rb, which in turn modulates E2F mediated transcription. However, the combination with palbociclib did not cause a complete growth inhibition and an enhanced senescence-like phenotype, instead it resulted in a prolonged quiescent state (Michaloglou et al., 2018).

In addition, a preclinical study assessing the effects of palbociclib and the mTOR inhibitor sapanisertib, showed that their combination synergistically inhibited breast cancer cell proliferation (Yamamoto et al., 2019).

## Implication for Treatments in Breast Cancer

The selection of systemic therapy in patients with advanced cancers is mainly related to HR and HER2 status, previous systemic therapies, and patient status. ER activity is effectively inhibited by endocrine agents. In addition, estrogen/ER complexes can also interact with AP-1 and SP-1 transcription factors which are able to modulate different transcriptional programs (Milla-Santos et al., 2003). On the other hand, the non-nuclear mechanism of action of ER, which can be bound to plasma membrane or free in the cytoplasm, consists in the activation of RTKs (Receptor Tyrosine Kinases) family receptors and, in turn, RTKs trigger their downstream pathways, such as PI3K/AKT/mTOR and Ras/MAPK (Giuliano et al., 2013).

The approval of modern CDK inhibitors has changed the treatment paradigm for advanced HR+ cancer. The use of palbociclib, abemaciclib and ribociclib, that are selective reversible inhibitors of CDK4 and CDK6, has been approved based on progression free survival benefit seen on phase III studies (Laderian and Fojo, 2017). Except for ER positivity, no other biomarkers predictive of response to CDK4/6 inhibitors have been identified so far.

### Molecular Mechanism of Resistance to CDK4/6 Inhibitors

Different molecular mechanisms of resistance to CDK4/6 inhibitors can arise (Pandey et al., 2019). They are due to p16, CDK6, CCNE1/2, CDK2, CDK4, or E2F amplification and also to loss of or mutations in Rb. It is remarkable that the loss of Rb is responsible for activation of E2F and the cyclin E-CDK2 axis, assuring progression of the cell cycle. Recently, it was elucidated that also loss of the FAT1 (FAT atypical cadherin 1) tumor suppressor can promote resistance to CDK4/6 inhibitors (Li et al., 2018). Therefore, there are different mechanisms of early resistance to CDK4/6 inhibitors that favor alternative pathways of S-phase entry.

CDK4/6 inhibitors or CDK4/6 inhibitor-resistant breast cancer cells activate the PI3K/AKT/mTOR pathway (Herrera-Abreu et al., 2016; Jansen et al., 2017; Pandey et al., 2019). mTOR promotes cell proliferation and growth by suppressing autophagy and promoting anabolic processes, such as biosynthesis of lipids and proteins. mTOR could have a more complex role since it also localizes in nucleus where it interacts with epigenetic regulators. A recent study by Michaloglou et al. (2018) suggests that, in CDK4/6 inhibitor-resistant ER-positive cancer cell lines, inhibition of mTORC1/2 restores sensitivity to CDK4/6 inhibitors (Michaloglou et al., 2018). Another investigation shows that PI3K inhibitors are able to prevent the resistance to CDK4/6 inhibitors. However, it fails to re-sensitize cells when resistance is acquired (Herrera-Abreu et al., 2016). According to the above mentioned *in vitro* studies, it seems better to hit mTOR first in order to avoid or delay tolerance to CDK4/6 inhibitors. Accordingly, in a small clinical trial including 23 metastatic HR+/HER2- breast cancer patients treated with palbociclib after pretreatment with everolimus, it has been reported a limited clinical benefit, since poor response rate and short PFS have been observed (Dhakal et al., 2018). Nevertheless, this data should be confirmed in randomized clinical trials comparing the sequences mTOR-CDK4/6 inhibitors vs. CDK4/6-mTOR inhibitors in patients with breast cancer.

It is important to note that this pathway reconstruction could have various limitations, including missing actors, unknown interactions and particular cases, such as gene amplifications or mutations that could cause the loss of function of some elements not taken into account.

### CDK4/6 Inhibitors in Combination With Other Therapies

The use of CDK4/6 inhibitors is being investigated in combination with other drugs, i.e., targeted therapy, immunotherapy and chemotherapy.

Pre-clinical data suggest that there is a synergistic effect when agents targeting the PI3K/mTOR pathway are combined with CDK4/6 inhibition. Preliminary information on a few patients has been observed when mTOR inhibitors (everolimus) and α-specific PI3K inhibitor (alpelisib) are used with abemaciclib and ribociclib. A phase Ib trial of ribociclib, everolimus, and exemestane in 83 pre-treated patients with HR+/HER2- advanced cancer showed an Overall Response Rate (ORR) of 13%. Twenty-three percent of the patients had received prior PI3K/AKT/mTOR inhibitors (Oliveira et al., 2016). The combined therapy was basically well tolerated and the safety profile was consistent with the combination of everolimus and exemestane. Furthermore, abemaciclib was investigated in combination with everolimus and exemestane in a small number of patients with HR+/HER2- metastatic cancer. Of the 15 patients that were evaluable for response assessment, the ORR was 33%, the clinical benefit rate at 6 months being 73%. Recently, metastatic breast cancer patients treated with the mTOR inhibitor everolimus, as first line therapy, have been subsequently treated with fulvestrant and palbociclib, resulting in a significantly better progression-free survival (Herrscher et al., 2019). Currently, there are several ongoing trials exploring combination of abemaciclib, ribociclib and palbociclib with agents targeting the PI3K/mTOR pathway, including pan-PI3K inhibitors (copanlisib), alpha-specific PI3K inhibitors (alpelisib, GDC-0077), PI3K/mTOR dual inhibitor (LY3023414), and mTOR inhibitors (everolimus).

It is reasonable to think that in some patients there is the possibility to restore sensitivity to CDK4/6 inhibitors. This could depend on which molecular mechanism is responsible for the resistance. These studies need to be further developed; in fact, they could reveal biomarkers to identify which patients will develop resistance and which, among them, can recover sensitivity to CDK4/6 inhibitors. Indeed, the identification of patients who develop resistance to CDK4/6 inhibitors due to the activation of PI3K/AKT/mTOR pathway will pave the way to the combination of abemaciclib, ribociclib or palbociclib with everolimus, alpelisib or copanlisib into daily clinical practice. This will represent a major step forward on the road to precision medicine in patients with advanced breast cancer.

## Conclusion

The reconstruction of pathways around estrogen receptor (ER), mTOR and cyclin D allowed the comparison of the effects of CDK4/6 and PI3K/AKT/mTOR inhibitors in metastatic HR+ cancer. Pre-treatment with mTOR pathway inhibitors could prevent or delay the onset of resistance to CDK4/6 inhibitors. Additional studies are needed in order to find the biomarkers that can identify patients who will develop this resistance and in whom the sensitivity to CDK4/6 inhibitors can be restored. Moreover, the combination of anti-mTOR/PI3K/AKT agents with CDK4/6 inhibitors should be further investigated basing on the results obtained in preliminary studies.

## Author Contributions

FP, MS, and AC: conception and design. GS and MC: acquisition of data. GO, MG, and FP: analysis and interpretation of data. GO and ER: drafting the manuscript. LC, GP, and RM: critical revision of the manuscript for important intellectual content. AM and NS: technical and material support. MG, NB, and FP: supervision.

## Conflict of Interest

The authors declare that the research was conducted in the absence of any commercial or financial relationships that could be construed as a potential conflict of interest.

## Footnotes

1 http://globocan.iarc.fr/Pages/fact_sheets_cancer.aspx
